# Twin reversed arterial perfusion (TRAP) sequence in association with VACTERL association: a case report

**DOI:** 10.1186/1752-1947-4-411

**Published:** 2010-12-22

**Authors:** Sharan Athwal, Katherine Millard, Kokila Lakhoo

**Affiliations:** 1 Department of Paediatric Surgery, John Radcliffe Hospital, Headington, Oxford, OX3 9DU UK

## Abstract

**Introduction:**

Twin reversed arterial perfusion (TRAP) sequence is a rare complication of multiple pregnancy caused by defects in early embryogenesis. The pump twin supplies the acardiac recipient twin with blood, and although the pump twin is usually structurally normal, congenital anomalies have sometimes been reported. We report a unique case of twin reversed arterial perfusion sequence with a prenatal diagnosis of VACTERL association in the surviving pump twin.

**Case presentation:**

A 24-year-old Caucasian woman presented at 11 weeks' gestation with a monochorionic, monoamniotic twin pregnancy. A reversed arterial flow was noted on a Doppler imaging study coming from the larger, apparently normal twin to the smaller, grossly abnormal twin, and a diagnosis of twin reversed arterial perfusion sequence was made. Cardiac activity was undetectable in the recipient twin by 16 weeks' gestation. Further detailed assessment at 18 weeks' gestation revealed multiple congenital anomalies of the surviving pump twin, in keeping with a diagnosis of VACTERL association. A live infant girl was delivered at 39 weeks by elective cesarean section. She underwent extensive surgery with subsequent normal development at the age of two years.

**Conclusion:**

The co-existence of two rare and complex conditions in this unique case raises interesting questions about the role of early defects in embryogenesis and their subsequent effects on fetal development. This case also highlights the importance of prenatal diagnosis of major congenital anomalies to the plan treatment, reduce morbidity and aid the survival of affected children.

## Introduction

Twin reversed arterial perfusion (TRAP) sequence is a rare complication of monozygotic multiple pregnancy. The presence of an acardiac twin occurs in one of every 35,000 twin pregnancies and in 1% of monochorionic twin pregnancies [1]. In cases of TRAP, the mortality rate for the normal (pump) twin is reported to be approximately 50% [2]. The majority of pump twins are congenitally normal, but anomalies are sometimes observed, including cardiogenic defects, gastroschisis and skeletal abnormalities [3].

VACTERL association is a relatively common, non-random complex malformation disorder with an incidence of 0.4-1.6 in every 10,000 births [4]. VACTERL association is classically defined as the presence of at least three of the following anomalies: vertebral defects (V), anal atresia (imperforate anus) (A), congenital heart lesions (C), tracheoesophageal defects (TE), renal and distal urinary tract anomalies (R) and limb lesions (L).

We report a case of a monochorionic twin gestation with TRAP sequence in which the surviving twin was diagnosed with VACTERL association.

## Case report

A 24-year-old gravida 3, para 2, Caucasian woman was referred to the fetomaternal medicine unit at 11 weeks' gestation with a monochorionic, monoamniotic twin pregnancy. The smaller twin (crown rump length [CRL] 22 mm, compared with 33 mm) appeared grossly abnormal with hydropic features. Two fetal heartbeats of different but normal rates were identified. Subsequent detailed assessment at 14 weeks' gestation revealed one twin to be of normal size and appearance, while the other twin had a head, an abnormal rudimentary heart, a trunk and two limbs only. A reversed arterial flow was noted on a Doppler imaging study coming from the apparently normal twin to the abnormal twin, and a diagnosis of TRAP sequence made.

By the 16-week scan, no cardiac activity or circulation was apparent in the abnormal twin. Further assessment at 18 weeks' gestation revealed that the surviving fetus had kyphoscoliosis of the upper lumbar spine with some abnormal vertebrae, a large outlet Ventricular Septal Defect (VSD) with aortic override and a small pulmonary artery. A provisional diagnosis of VACTERL association was made. A live Caucasian girl, birthweight 3324 g, was delivered at 39 weeks' gestation by elective Lower Segment Caesarean Section (LSCS). Apgar scores were 9 and 10 at one minute and five minutes, respectively. The monochorionic, monoamniotic placenta weighed 685 g, and only two cord vessels were present. An abnormally formed fetus measuring 3.8 cm was also delivered, and two legs were identified.

Postnatally, the surviving twin was found to have tetralogy of fallot, a high anorectal malformation with rectovestibular fistula, ureteric and bladder abnormalities causing reflux and incomplete emptying, and lumbosacral abnormalities. A split colostomy was formed on day two, which was later closed and reformed after complications. The patient also underwent vesicostomy and balloon pulmonary valvotomy. A follow-up examination showed normal development at two years of age.

## Discussion

Chorioangiopagus parasiticus or TRAP syndrome is the most extreme manifestation of twin-twin transfusions. Usually, one twin has acardia (the recipient) and the other is structurally normal (the donor or pump twin). The pump twin supplies deoxygenated blood via vascular anastomoses to the acardiac twin. This blood flow is characterized by reversed arterial perfusion (hence twin reversed arterial perfusion).

The pathogenesis of TRAP is controversial, with two main pathogenetic hypotheses proposed [5]:

1. Deep placental anastomoses in early embryogenesis cause malformation of the acardiac twin. The early pressure flow in one twin exceeds that of the other and leads to reversed circulation in the twin who exhibits perfusion.

2. A primary defect in embryogenesis in one twin leads to failure of cardiac development. The normal twin then perfuses the acardiac twin via artery-artery anastomoses. However, the anastomoses are not responsible for the cardiac anomaly.

The mortality of the acardiac twin is 100%, and the perinatal mortality of the pump twin is reported to be around 50%. The poor outcome of the pump twin is primarily due to congestive heart failure, cord entanglement or prematurity [6]. The mortality rate of the pump twin appears to correlate with the size of the acardiac twin. A larger, more developed acardiac twin (with multiple internal organs such as kidneys) is associated with a significant increase in the perinatal mortality of the pump twin [7]. Other poor prognostic indicators are polyhydramnios and preterm delivery (before 32 weeks' gestation).

Although the pump twin is usually structurally normal, there is an incidence of around 10% of major malformations [5]. There is an increased frequency of structural defects in association with monozygotic twinning. Jones and Benirschke [8] reported that some structural defects noted in a monozygotic twin pair were caused by an intrauterine vascular accident. He suggested that transfer of thromboplastin-rich blood from a dead to a living monozygotic twin through placental anastomoses led to embolization and infarction, resulting in defects in major organ systems. Recently, the more accepted hypothesis is one of hemodynamic imbalances with blood pressure chances and possible blood loss from the normal to the abnormal twin [9].

Our case is unique in the literature, as the pump twin was found to have VACTERL association prenatally. The acronym VATER association was originally proposed in 1972 [10], and subsequently it was expanded to include cardiac and renal defects. To diagnose VACTERL association, the fetus or infant must have three or more of the classic anomalies (as outlined in the Introduction above). The most commonly associated anomaly has been found to be congenital heart disease (32-50%) [11]. The wide spectrum of defects in VACTERL association suggests an alteration early in embryogenesis. Although prenatal diagnosis of this condition is not always possible, ultrasound and magnetic resonance imaging (MRI) can allow the clinician to visualize some of the characteristic findings, as in our case.

## Conclusion

The prenatal diagnosis of both TRAP sequence and VACTERL association are important to aid survival. Early diagnosis of TRAP sequence enables both invasive and conservative treatment to be offered to the mother to reduce the amount of compromise of the pump twin [12]. Infants with VACTERL association require significant surgical treatment and planning for delivery, and care of the neonate is vital. This case highlights the association between TRAP sequence and congenital anomalies in monozygotic twins and the role of probable defects in early embryogenesis. It also demonstrates that prenatal diagnosis and timely management can aid the normal development of a child who has rare and complicated congenital anomalies.

## Consent

Written informed consent was obtained from the guardians of the patient for publication of this case report.A copy of the written consent is available for review by the Editor-in-Chief of this journal.

## Competing interest

The authors declare that they have no competing interests.

## Authors' contributions

KM presented the case history, researched the topic and helped draft the manuscript. SA reviewed the literature and drafted the manuscript. KL was the supervising consultant who performed the surgery, obtained consent for publication and edited the manuscript. All authors read and approved the final manuscript.
